# Antioxidant N-acetyl-L-cysteine ameliorates symptoms of premature aging associated with the deficiency of the circadian protein BMAL1

**DOI:** 10.18632/aging.100113

**Published:** 2009-12-30

**Authors:** Roman V. Kondratov, Olena Vykhovanets, Anna A. Kondratova, Marina P. Antoch

**Affiliations:** ^1^ Department of Biological, Geological and Environmental Sciences, Cleveland State University, Cleveland, OH 44115, USA; ^2^ Departments of Cancer Biology Cleveland Clinic Foundation, Cleveland, OH 44195, USA; ^3^ Departments of Molecular Genetics, Cleveland Clinic Foundation, Cleveland, OH 44195, USA; ^4^ Department of Molecular and Cellular Biology, Roswell Park Cancer Institute, Buffalo, NY 14263, USA; ^5^ Present address: Department of Urology, Case Western Reserve University, Cleveland, OH 44106, USA

**Keywords:** aging, oxidative stress, circadian clock, BMAL1

## Abstract

Deficiency of the circadian clock protein BMAL1 leads to premature aging and increased levels of reactivate oxygen species in several tissues of mice. In order to investigate the role of oxidative stress in accelerated aging and development of age-related pathologies, we continuously administered the antioxidant N-acetyl-L-cysteine toBmal1-deficient mice through their entire lifespan by supplementing drinking water. We found that the life long treatment with antioxidant significantly increased average and maximal lifespan and reduced the rate of age-dependent weight loss and development of cataracts. At the same time, it had no effect on time of onset and severity of other age-related pathologies characteristic of Bmal1-/- mice, such as joint ossification, reduced hair regrowth and sarcopenia. We conclude that chronic oxidative stress affects longevity and contributes to the development of at least some age-associated pathology, although ROS-independent mechanisms may also play a role. Our bioinformatics analysis identified the presence of a conservative E box element in the promoter regions of several genes encoding major antioxidant enzymes. We speculate that BMAL1 controls antioxidant defense by regulating the expression of major antioxidant enzymes.

## INTRODUCTION

The circadian clock is a universal time keeping system that generates 24-hr rhythms in behavior and physiology. The activity of the circadian system is important for synchronization of metabolic processes within an organism and between an organism and its environment [1,2]. The importance of this coordination for human health is supported by a number of epidemiological studies demonstrating that the risk of many diseases, including cardiovascular disease and cancer, is significantly increased among shift workers; however, the exact mechanisms linking circadian desynchronization and the development of various pathological conditions remains largely unknown [3]. At the molecular level the activity of the circadian clock is controlled by several interlocked transcription/translation feedback loops formed by the core circadian proteins [4,5]. Mice with a targeted disruption of different circadian proteins lose rhythmic patterns of behavior and develop multiple physiological abnormalities [3,6]. Recently, a connection between the circadian clock and aging has been established. It is most prominently manifested in mice deficient in the BMAL1 protein. During the normal course of their life, these animals develop multiple pathological changes that are characteristic of premature aging [7]. This is in sharp contrast to other circadian mutant mice models, such as *Clock/Clock *and*Per2^m/m^*animals, which accelerate their aging program and develop phenotypes that are reminiscent of those in *Bmal1*-deficient mice only after being exposed to a low dose of ionizing radiation [8,9].

BMAL1 is a basic helix-loop-helix (bHLH)-PAS domain transcription factor and a key component of the circadian clock [10]. Deficiency in the BMAL1 protein results in disruption of rhythmicity in behavior and gene expression pattern [11]. BMAL1 is involved in the control of tissue homeostasis by the direct regulation of reactive oxygen species (ROS); accordingly, its deficiency is associated with the excessive production of ROS resulting in chronic oxidative stress [7]. Many life-threatening diseases, including cardiovascular disease, cancer and diabetes, have been linked to chronic oxidative stress [12]. It has also been proposed that oxidative stress plays an important role in the development of age-associated pathology [13,14]; however, many aspects regarding its exact role in the process of aging are still under debate [15].

Previously we have shown that age-related degenerative processes in several tissues of *Bmal1-/-* mice are correlated with an age-dependent increase in the level of ROS [7]. If excessive production of ROS and increased oxidative stress contribute to the early aging phenotype in *Bmal1-/-* mice, then the reduction of oxidative stress by antioxidants might prevent early aging or ameliorate its severity. This strategy has been previously successfully used to delay ROS-initiated degenerative processes in nematode, fly and mouse [16].  Among available antioxidants, a potent low molecular weight (LMW) antioxidant N-acetyl-L-cysteine (NAC) was proved to be efficient in mice; indeed, treatment with NAC significantly delayed tumorigenesis in *p53-/-* mice [17] and ameliorate age-related pathological changes induced by the deficiency of transcription factor FOXO [18]. Here we confirm the role of chronic oxidative stress in early onset of aging in *Bmal1-/-* mice. We show that continuous administration of NAC delays the onset of aging and extends the lifespan of *Bmal1*-deficient mice. We speculate that BMAL1 controls antioxidant defense by regulating the expression of major antioxidant enzymes.

## RESULTS

## NAC slows age-dependent body weight loss in BMAL1-deficient mice

To investigate the effect of antioxidants on age-dependent weight loss in BMAL1-deficient mice, we started treating the experimental animals from the time of their prenatal development by supplementing drinking water of the breeders with NAC. To generate age-matched control animals, similar breeding pairs, which were set up simultaneously, received regular water. The prolonged administration of NAC had no effect on the size of litters born or on the survival of pups during lactation. After weaning, the animals in the experimental group received water supplemented with NAC through their entire lifespan.

**Figure 1.**
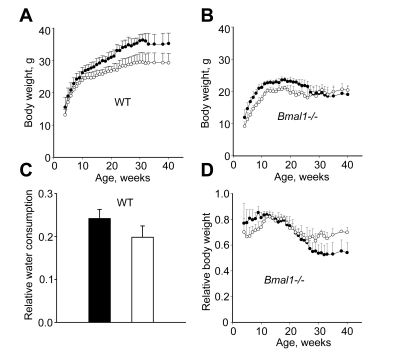
Continuous administration of NAC affects age-dependent changes in body weight. Total body weight of male (**A**) WT and (**B**) *Bmal1-/-* mice, closed circles - control mice raised on regular water; open circles - mice raised on water supplemented with 40mM of NAC. (**C**) Relative water consumption by WT mice receiving either regular (closed bar) or NAC-supplemented (open bar) water. (**D**) Age-dependent changes in relative body weight in *Bmal1-/-* mice measured at each time point as the percentage of the body weight of WT mice of the same group.

We started monitoring the body weight of wild type (WT) and *Bmal1-/-* mice from 4 weeks of age. The administration of NAC slows down age-dependent body weight gain in mice of both genotypes (Figure 1A,B). Thus, by the time the body weight of WT mice normally reaches its maximum (30 weeks of age) and stabilizes, animals receiving NAC weigh ~18% less than their littermates raised on regular water (Figure 1A). Similarly, until reaching their maximal weight (at 18 weeks), *Bmal1*-deficient mice that received NAC weigh less than the corresponding control animals drinking regular water (23.6+ 0.84 and 21.15+ 0.55 g respectively, Figure 1B, p<0.01). To test whether this effect could be attributed to taste preferences, we measured daily levels of water consumption in both groups of WT mice and in fact determined that mice that receive NAC drink significantly less water (Figure 1C). To account for these differences in consumption, we compared the effect of NAC on the relative weight of BMAL1-deficient animals (measured at each time point as % of the body weight of WT mice of the same group (Figure 1D). As shown in Figure 1D, starting from 25 weeks of age, when *Bmal1-/-* mice normally begin losing weight [7], animals raised on NAC-supplemented water have significantly higher relative weight than animals in the control group that received regular water. As a result, at 40 weeks of age *Bmal1-/-* mice in the control group lost on average 20% of their maximal body weight; whereas the body weight of animals that received NAC was reduced by 4%.  Thus, treatment with the LMW antioxidant NAC significantly delayed age-dependent weight loss in BMAL1*-*deficient mice.

## Effect of NAC on development of the phenotype of premature aging in *Bmal1-/-* mice.

Previously we have demonstrated that the deficiency of BMAL1 is associated with an early onset of several phenotypes associated with normal aging [7]. Among those are reduced hair regrowth after shaving, development of cataracts, cornea inflammation, sarcopenia and joint ossification. This prompted us to test whether the administration of NAC affects the onset and/or severity of these changes.

**Figure 2.**
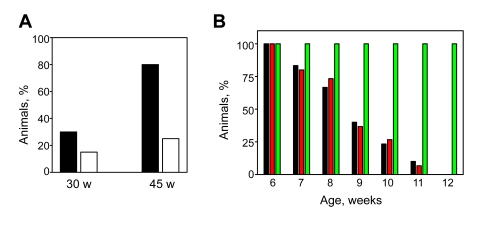
Effects of continuous administration of NAC on development of eye pathology and muscle strength. (**A**) Frequency of cataracts in 30-week old and 45-week old *Bmal1-/-* mice raised on regular (closed bars) or NAC-supplemented (open bars) water. Each eye was counted independently; the percentage of cataracts was determined by dividing the number of cataracts by the total number of eyes, if an animal was dead at the time of observation, then the previous score for this animal was used. (**B**) Age-dependent changes in muscular strength of WT (green bars) and *Bmal1-/-* mice receiving regular (black bars) or NAC-supplemented (red bars) water. Muscular strength was evaluated as the ability of animals of indicated age to maintain their weight on the inverted grid. Each animal was tested five times, if the animal did not fall down for 30 sec the trial was counted as successful. The percentage of successful trials was calculated and plotted. No difference was detected between NAC-treated and control *Bmal1-/-* animals.

One of the most striking age-dependent changes related to deficiency in BMAL1 is the early onset of various eye pathologies, such as cataracts and cornea inflammation [7]. At 30 weeks of age, *Bmal1-/-* animals in both groups start showing various degrees of eye pathologies with a slightly higher incidence in the control group (30% versus 15% in NAC-receiving animals). The difference between the two groups increased with age; 80% of 45-week old control mice developed cataracts on one or both eyes, whereas in the NAC-treated group only 25% of animals were affected (Figure 2A).

The comparison of the two groups for the severity of other hallmarks of aging did not reveal any significant differences. Thus, the administration of NAC did not improve reduced hair regrowth characteristic of BMAL1-deficient mice: only 3 out of 10 NAC-treated mice de-monstrated partial or complete hair regrowth after shaving, which was not different from controls (4 out of 10).

Treatment with NAC had no effect on the development of joint ossification evaluated by changes in ankle joint flexibility and physical performance.  The latter was estimated by measuring righting reflex time (the time required for mice to return to their normal position after being placed on the back).  Whereas young WT and *Bmal1-/-* mice normally take less than 1 sec, up to 3 sec was required for 30-week old *Bmal1-/-* mice, regardless of NAC supplementation.

In order to estimate the effect of antioxidants on the aging of muscles, we measured grip strength by monitoring the ability of mice in both groups to maintain weight on the inverted grid. Performance of WT mice in this task did not change during their lifespan, whereas in *Bmal1-/-* mice it was gradually reduced with age (Figure 2B). However, administration of NAC did not improve the age-related decrease in muscle strength. Thus, administration of the LMW antioxidant NAC significantly delayed age-related development of cataracts, but had no effect on manifestation of other pathological changes in *Bmal1-/-* mice associated with premature aging.

## Continuous administration of NAC extends lifespan of *Bmal1-/-* mice

**Figure 3.**
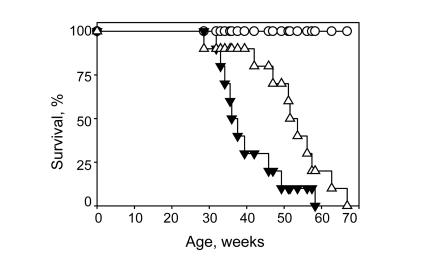
Continuous administration of NAC increases lifespan of *Bmal1-/-* mice. Kaplan-Meyer survival curves were obtained for WT mice raised on regular (closed circles) or NAC-supplemented (open circles) water; and *Bmal1-/-* mice raised on regular (closed triangles) or NAC-supplemented (open triangles) water. NAC significantly increased lifespan of *Bmal1-/-* mice (P =0.022, log-rank Mantel-Haenszel test).

Consistent with our previous report, *Bmal1*-deficient mice in the control group had a very short average lifespan of 38+ 11 weeks. As shown in Figure 3, continuous administration of NAC extends the lifespan to 47+ 12 weeks (p<0.05 Log-Rank Test). The survival curve for NAC-treated mice was significantly shifted, with 90% survival at the age of 36 weeks (only 50% of animals survived until this age in the control group).  Treatment with the antioxidant also significantly affects the maximum lifespan, extending it from 58 weeks in the control group to 66 weeks in the NAC-treated group. All wild type mice in both the control and NAC-treated groups survived until the termination of the experiment (70 weeks). Thus, treatment with a dietary antioxidant increases the average lifespan in *Bmal1-*deficient animals by about 24% and maximum lifespan by 14%.

## Genes encoding major antioxidant enzymes are potential targets of the CLOCK/BMAL1 transcrip-tional complex

Our current and previous results led us to the hypothesis that BMAL1 may be involved in the control of an organism's response to oxidative stress and antioxidant defense. Antioxidant defense is controlled by a complex system of LMW antioxidants and antioxidant enzymes [12]. As a transcription factor working in complex with CLOCK or NPAS2, BMAL1 may regulate the activity of major antioxidant enzymes (MAE) at the transcriptional level. CLOCK/BMAL1 and NPAS2/ BMAL1 complexes specifically bind promoters containing circadian E box in their regulatory regions.

**Table 1.** Position of the circadian E-box elements in the promoter regions (+/- 2000 nucleotides from major transcription starting site) of genes encoding major antioxidant enzymes. SOD - Superoxide dismutase; CAT - catalase;  GPX - glutathione peroxidase; PRDX - peroxiredoxin; TXNRD - thioredoxin reductase; SESN - sestrin

	**Homo Sapiens**	**Pan Troglodites**	**Macaca Mulatta**	**Mus Musculus**	**Rattus Norvegicus**
**SOD1**	-886	-1055	-1044	-631, 768, 1787	-1661, -685, 743, 1749
**SOD2**	none	n/a	n/a	none	none
**SOD3**	-1673	n/a	n/a	none	none
**GPX1**	-19	-23	-93	-1196, -908, -54, 12	-82
**GPX2**	974	n/a	n/a	1263	72
**GPX3**	979	1068	1061	-570, 1268	-1507, 198, 921
**GPX4**	-385	n/a	n/a	-1669	none
**GPX6**	173	n/a	n/a	-617, -168, 1580	none
**CAT**	-1751	none	413	46, 1437	-969, 47, 1434
**PRDX1**	372	none	407	218, 528	-1413, -1402, 198
**PRDX2**	none	n/a	n/a	none	none
**PRDX3**	-136	-142	-148	428	406, 1733
**PRDX4**	none	n/a	n/a	none	none
**PRDX5**	-836	n/a	n/a	-1019, -282	none
**PRDX6**	-290, 991	-852, 474	-229, 1048	-159, -114, 264, 904	-185, -140, 248
**SESN1**	345, 1089	464	-1549, -733	-292, -776, 1390	-759
**TXNRD1**	-260	n/a	n/a	1510	-841, 170, 975

Two circadian E box elements have been identified: CACGTG and CACGTT [19].  To test if any of the MAE genes can be directly regulated by the major circadian transactivation complex, we performed *in silico* analysis of their promoter regions for the presence of BMAL1-responsive elements. Nucleotide sequences covering the region between -2000bp/+ and 2000bp (relative to the position of the transcriptional start site) of the NCBI database were analyzed using EditSeq and MegAlign software (DNASTAR, Inc.). The results of the analysis summarized in Table 1 indicate that many of the MAE genes may in fact be directly regulated by the CLOCK/BMAL1 transcriptional complex.  Most strikingly, the position of the BMAL1-responsive elements in the promoters of several MAE genes such as Gpx1, Prdx1, Prdx6, and Sesn2 is conservative among primates and rodents, indicating their potential functional significance.

## DISCUSSION

The free-radicals theory of aging postulates that oxidative damage to biological macromolecules produced by ROS and RNS play an important role in the aging process [13,14,20]. This theory is supported by the large amount of experimental data demonstrating a direct correlation between the resistance to oxidative stress and the lifespan in different organisms [16,21]. However, this theory was recently challenged by contradictory data obtained in various mouse models demonstrating that although the overexpression of several antioxidant enzymes makes mice more resistant to oxidative challenge, it fails to increase their lifespan. Thus, the deficiency of superoxide dismutase reduced the lifespan in mice, whereas the deficiency of other antioxidant enzymes had no effect [22]. At the same time, targeted overexpression of catalase in mitochondria results both in reduced oxidative damage in tissues of transgenic mice and an increased lifespan [23]. Such conflicting data may arise from the fact that laboratory mice are normally maintained under optimal husbandry conditions, their antioxidant defense is well balanced and works efficiently in protecting from relatively low levels of ROS, therefore overexpression of antioxidant enzymes has a marginal effect. At the same time, the disruption of the antioxidant defense will have a more dramatic effect on the lifespan and may significantly contribute to the development of age-associated pathologies.

ROS and RNS are produced in the organisms either as side products of metabolic reaction or by a specific group of enzymes. ROS and RNS serve as important mediators of intra- and extra- cellular signaling and many physiological processes are regulated by specific species [12].  An excessive amount of ROS results in damage to biological macromolecules, which is known as oxidative stress and is an essential contributor to the development of such diseases as cancer, diabetes and cardiovascular diseases [12]. Therefore, ROS and RNS levels are tightly controlled at both intra- and extra-cellular levels by antioxidant systems. Previously we have demonstrated that accelerated aging of *Bmal1-* deficient mice is associated with an age-dependent increase in the level of ROS in different tissue [7]. The fact that an increase in ROS concentration was detected in those tissues that demonstrate pathological changes may suggest that the early onset of aging in *Bmal1*-deficient mice is caused by excessive production and/or insufficient detoxification of ROS.  Here we show that continuous administration of antioxidant NAC can significantly ameliorate the onset and severity of premature aging in *Bmal1*-deficient mice. Thus, deregulation of ROS homeostasis in fact contributes significantly to the premature aging phenotype initiated by the deficiency of the BMAL1 protein.

Noteworthy, treatment of *Bmal1-/-* mice with NAC cannot completely prevent premature aging; growth retardation, reduced hair regrowth, sarcopenia, and joint ossification were not affected by administration of NAC. There are two possible explanations for the incomplete rescue. First, NAC treatment may not be efficient enough due to tissue-specific differences in its distribution, which may restrict the antioxidant effect to a particular tissue. Second, BMAL1 may be involved in the control of aging through both ROS- dependent and ROS-independent mechanisms. However, administration of NAC attenuated the development of the most prominent age-related phenotype of *Bmal1-/-* mice, development of cataracts, and even most importantly, significantly extended the average and maximal lifespan of *Bmal1-/-* mice.

BMAL1 is a transcription factor critical for circadian function. In complex with its dimerization partners, CLOCK or NPAS2, BMAL1 controls the expression of several clock genes and multiple clock-controlled genes (CCGs). Based on microarray data, the about 10% of all transcripts display daily oscillations in expression, indicating that they may be clock-regulated [24]. The results of the bioinformatics analysis of the promoter regions of several genes encoding major antioxidant enzymes reveal the presence of conservative circadian E box elements, suggesting that at least some of the genes encoding antioxidant enzymes can be CCGs. Importantly, potential targets of BMAL1 include antioxidant enzymes, which control different stages of ROS detoxification. Among those are superoxide dismutase that converts superoxide into hydrogen peroxide; catalase, peroxiredoxines and glutathione peroxidase that reduce hydrogen peroxide and sestrins that are key regulators of oxidized peroxiredoxins reduction. Therefore, by controlling different steps of the process, the CLOCK/BMAL1 transcriptional complex may orchestrate the entire chain of reduction/oxidation reactions, which are necessary for the efficient detoxification of ROS.

The importance of circadian orchestration of antioxidant defense is supported by the fact that the disruption of this control results in oxidative stress leading to various pathological developments. Supporting this hypothesis are epidemiological data on disease spectra in shift workers. It is documented that disturbance of the circadian system through shift work or frequent travel across time zones leads to increased risk of cardiovascular diseases, diabetes and cancer. Although the molecular pathways responsible for this link are mostly unknown [25-27], it is well accepted that oxidative stress is one of the major causes in pathophysiology of these diseases. We speculate that when BMAL1-dependent circadian control of the antioxidant defense of an organism is disrupted by shift work, it leads to oxidative stress and increases risk of disease.

In summary, we demonstrated that treatment with the LMW antioxidant NAC delivered as a dietary supplement ameliorated the aging of BMAL1 deficient mice. These results suggest that an increased level of ROS is involved in the development of accelerating aging in this animal model. BMAL1 may control the ROS level through regulation of expression of major antioxidant enzymes, some of which are potential transcriptional targets of the CLOCK/BMAL1 complex. While circadian control of ROS homeostasis is critical for aging, some other oxidative stress independent mechanisms may also be involved.

## Experimental procedures

Animals. *Bmal1-/- *mice that were originally obtained from Dr. Bradfield (University of Wisconsin) were backcrossed to C57BL/6J mice for 12 generations. The colony was maintained as a heterozygous intercross to obtain animals of all three genotypes. Mice were genotyped by PCR as previously described [11]. All animals were maintained on a 12 h:12 h light:dark cycle in standard plastic cages and lifespan was determined by recording the age at spontaneous death. Animals treated with the antioxidant received 40mM NAC in drinking water during their entire life, starting from prenatal development (breeding pairs were maintained on NAC); water bottles were changed once every three days.  To monitor body weight gain/loss, animals were weighed once a week. Mice were observed daily for the general health status and to score mortality. Each group was represented by 10 animals.

Hair regrowth assay. Was performed on 30-week old mice as previously described [7]. Dorsal segments of skin were shaved and animals were monitored for hair regrowth for 3 months.

Estimation the muscle strength . Animals were placed on a wire cage top, which then was gently flipped over. Each animal was tested in five trials; a trial, in which animal did not fall down for 30 sec was scored as successful and the percentage of successful trials was calculated.

Righting reflex. Mouse was turned over onto its back and the time necessary to return back to a normal position (i.e. to right itself onto all four feet) was measured. Each measurement was performed five times for each mouse.

Detection of cataracts . Eye opacity was evaluated and scored under bright light by two independent experienced observers, who were blind to treatment and genotype. Every eye was counted independently; therefore the percentage was determined by dividing the number of cataracts by the total number of affected eyes. If an animal was dead at the time of observation, the previous score was added to the total number. Cataracts of different severity were score equally. All animal studies were conducted in accordance with the regulations of the Committees of Animal Care and Use at the Cleveland Clinic Foundation, Cleveland State University and Roswell Park Cancer Institute.

Statistical analyses. All statistical analyses were performed using SigmaStat 3.5 software (Systat Software, Inc., CA). Lifespan curves were calculated using Kaplan-Meier survival analysis; the statistical significance of curves was assessed using log-rank Mantel-Haenszel tests. P values <0.05 were considered as significant; the median, mean, and maximum survivals were calculated for each group.

### ACKNOWLEDGEMENTS

We thank Dmitry Gudkov for the editorial help. This work was supported by NIH grants CA102522 and GM075226 to M.P.A and AHA grant 0835155N to R.V.K.
